# Hand Hygiene and Antimicrobial Resistance in the COVID-19 Era: An Observational Study

**DOI:** 10.3390/antibiotics12030583

**Published:** 2023-03-15

**Authors:** Antonino Russotto, Edoardo Rolfini, Giovanni Paladini, Claudia Gastaldo, Costanza Vicentini, Carla Maria Zotti

**Affiliations:** Department of Public Health and Paediatrics, University of Turin, 10126 Turin, Italy

**Keywords:** hand hygiene, COVID-19, Italy, infection control, healthcare-associated infections, antimicrobial resistance

## Abstract

Hand hygiene (HH) is one of the most important infection prevention and control strategies at the hospital level. The aim of this study was to evaluate the potential COVID-19 pandemic impact on HH practices and rate of healthcare-associated infections. Data on alcohol-based handrub consumption (AHC) and antimicrobial resistance across 27 Italian hospitals over the period 2017–2021 were considered. Data on Methicillin-resistant *Staphylococcus aureus* (MRSA) and carbapenem-resistant Enterobacteria (CRE) were extracted from the antimicrobial resistance regional surveillance system. A significant increase was highlighted, with a peak in 2020 and a partial fall in 2021 for AHC (*p* < 0.001). The decrease in MRSA rates in 2021 compared to 2017–2019 was significant (*p* = 0.013). A significant Spearman’s correlation between AHC and CRE rates was found (Spearman’s ρ −0.646, *p* = 0.032). This study supports the importance of AHC monitoring and showed that improving AHC was an attainable goal in the COVID-19 era. However, other strategies are needed to maintain the high levels of AHC attained during the pandemic, in order to avoid a progressive drop that has already begun in 2021. Furthermore, our results support the inverse relationship between AHC and infection rates and antimicrobial-resistant bacteria.

## 1. Introduction

Hand hygiene (HH) is recognized as one of the most important prevention strategies in reducing the transmission of pathogens between and among health-care workers (HCWs) and patients.

HH is an essential measure in reducing healthcare associated infections (HAIs) and for preventing the spread of multidrug-resistant organisms (MDRO) [1,2]. Both HAIs and antimicrobial resistance (AMR) are an important risk for patient safety [3]. Several studies have shown HAIs and AMR increase morbidity, mortality, healthcare costs [1,4], and are associated with a significant burden of disease [5,6,7]. National and international institutions, including the US Centers for Disease Control and Prevention (CDC) and the World Health Organization (WHO), have highlighted the importance of HH. The CDC produced its own HH guidelines in 2002 [8], while the WHO promoted the global ‘Clean care is safer care’ HH campaign in 2005 [9] and released its “Hand Hygiene Guidelines in Health Care” in 2009 [4]. Moreover, the WHO outlined a multimodal strategy to improve HH adherence among healthcare workers [2,10]. The use of alcohol-based products for routine HH in healthcare facilities represents a valid alternative to the traditional use of soap and water (hand washing), both in terms of effectiveness in eliminating microorganisms and dermatological tolerability [11,12], with the following exceptions: if hands are visibly dirty and/or contaminated, or after exposure to potential spore-forming pathogens [4].

The appropriateness of HH practices can be assessed in several ways. The direct method involves observation by a specialized operator and is considered the gold standard for hand hygiene monitoring. This method allows evaluating the behavior of single HCWs. Another strategy is the indirect method, i.e., measuring alcohol-based handrub consumption (AHC). This method requires less time and resources than direct observation [13]. Monitoring AHC though surveillance programs allows the identification of specific hospitals, departments, or wards where quality improvement strategies should be targeted [14].

During the COVID-19 pandemic, HH and personal protective equipment (PPE) proved essential measures to avoid the spread of SARS-CoV-2 among patients and HCWs, considering the main transmission mechanisms for the virus are transmission via direct contact and droplets [15]. During the pandemic, assessing HH compliance through direct observation proved difficult, as infection prevention and control (IPC) resources were diverged to emergency management, while isolation hindered direct observation. Notwithstanding these difficulties, several studies carried out in acute care hospitals suggest HH compliance increased significantly during the pandemic compared to previous years; however, this trend was not maintained in the longer term [3,14,16,17].

In Piedmont, a region in Northern Italy, since 2014, AHC in acute-care facilities is monitored as part of a wider HAI and IPC regional quality indicator surveillance program. In 2020, the region joined a national surveillance system for AHC, coordinated by the National Health Institute (Istituto Superiore di Sanità, ISS) [18]. The purpose of this study was to describe trends in AHC and AMR at the regional level from 2017 to 2021, in relation to the COVID-19 pandemic, to identify areas for improvement and a potential relationship between the two variables.

## 2. Results

**Figure 1:**
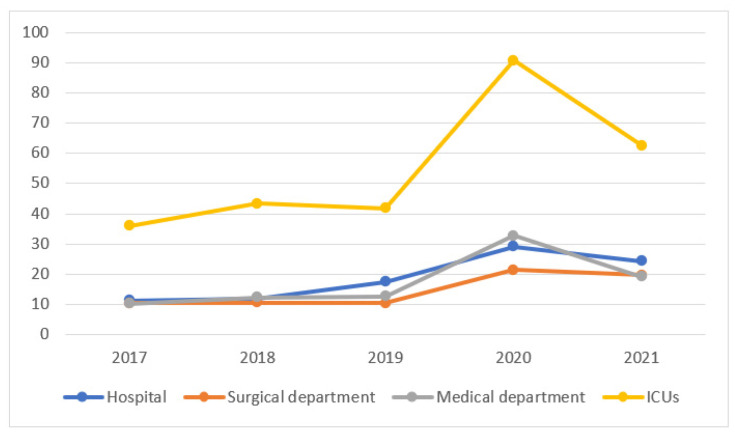
Median alcohol-based handrub consumption (AHC, liters/1000 patient-days) among 27 hospitals in Piedmont, 2017–2021.

**Figure 2:**
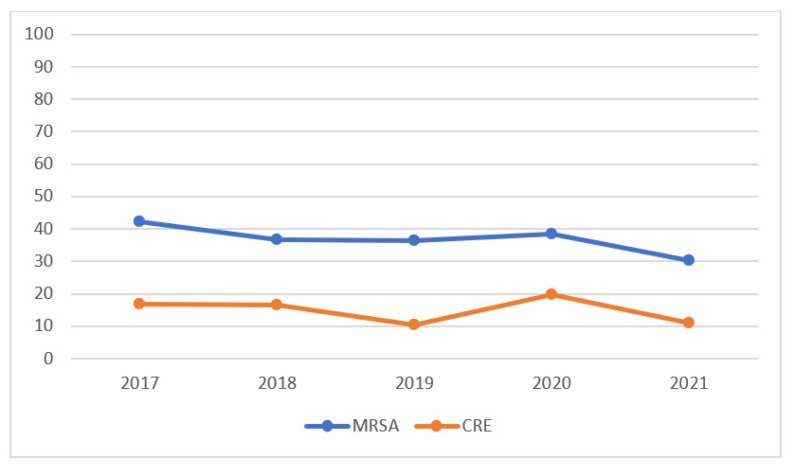
Median methicillin-resistant Staphylococcus aureus (MRSA) and carbapenem-resistant Enterobacteria (CRE) rates among invasive isolates from 27 hospitals in Piedmont, 2017–2021.

Regional-level AHC, MRSA, and CRE trends are represented in Figure 1 and Figure 2. Figure 1 shows median AHC as liters per 1000 patient-days (l/1000 pd), overall and according to department type. As shown in the Figure 1, hospital-level ACH saw a steady increase from an initial 11.3 l/1000 pd in 2017, with a peak of 29.2 l/1000 pd in 2020, followed by a fall to 24.4 l/1000 pd in 2021. This was mainly influenced by AHC at the ICU-level, which from an initial 36.1 l/1000 pd in 2017 increased to 90.7 l/1000 pd in 2020, and decreased to 62.6 l/1000 pd in 2021.

Figure 2 shows median MRSA and CRE rates. While no visible trend was identifiable for CRE, with infection rate peaking in 2020 (19.8%), the MRSA trend showed a progressive decrease, going from 42.3% in 2017 to 30.4% in 2021. However, the decreasing trend was not significant at Mantel–Haenszel extended Chi-square testing for a linear trend (X^2^ = 2.17, *p* = 0.141).

Table 1 reports median AHC (overall and according to department type), as well as overall median MRSA and CRE rates at the hospital level per considered period. Concerning AHC, a significant two- to three-fold increase was found comparing 2020 median data to 2017–2019 median data, at all considered levels. A significant increase compared to 2017–2019 was also found considering 2021 data; however, AHC was lower compared to 2020 at all considered levels. No significant difference was found between MRSA and CRE infection rates in the period 2017–2019 compared to 2020 and 2021, respectively, except for a significant decrease in MRSA rates in 2021 compared to 2017–2019.

Results of the time-repeated Spearman correlation analyses between AHC and MRSA/CRE rates are summarized in Table 2. Due the lack of data of MRSA/CRE rates, the correlation was not performed for all the facilities. In the years 2020 and 2021, all correlations had the same direction, i.e., an inverse proportion between AHC and infection rates was found. A moderate significant correlation was found between AHC and CRE rates at the hospital level in 2021.

**Figure 3:**
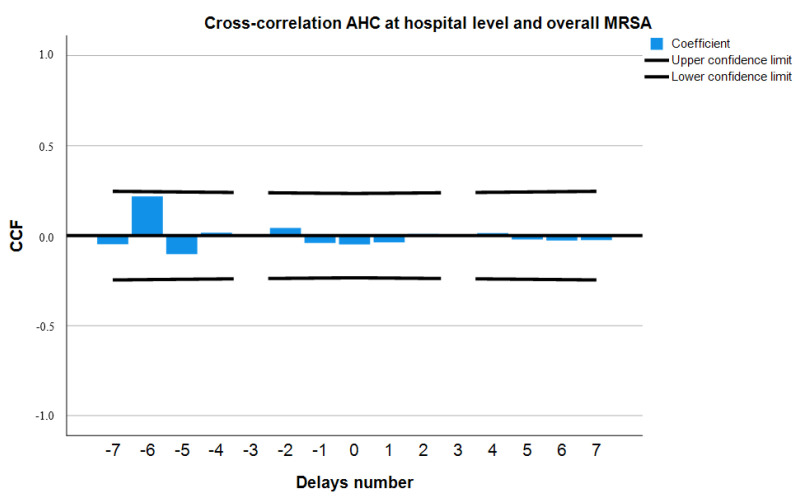
Cross−correlation between alcohol-based handrub consumption (AHC) and methicillin-resistant Staphylococcus aureus (MRSA) rate at the hospital level.

**Figure 4:**
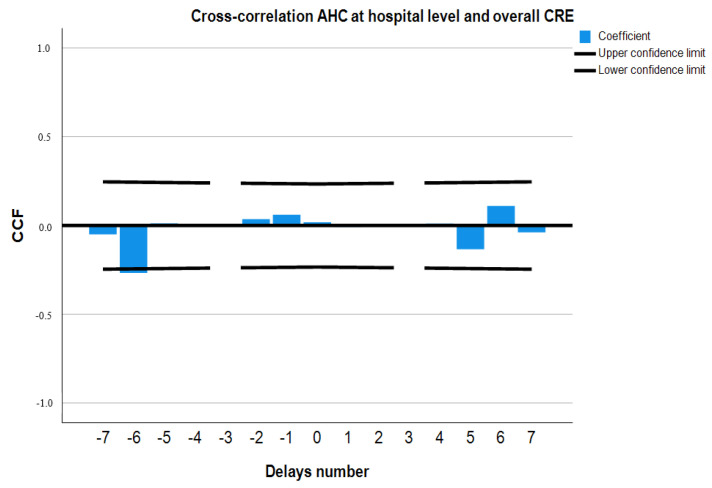
Cross−correlation between alcohol-based handrub consumption (AHC) and carbapenem-resistant Enterobacteria (CRE) rate at the hospital level.

Two cross-correlation analyses were performed, correlating AHC at the hospital level with overall MRSA and CRE rates (Figure 3 and Figure 4). As shown in the figures, no significant differences emerged.

## 3. Discussion

This study evaluated AHC and AMR rates for MRSA and CRE infections over a 5-year period, from 2017 to 2021, in the Piedmont region. We investigated the possibility of a relationship between the two variables and performed a comparative evaluation between the pre- and post-pandemic period.

In line with previous reports [3,14,16], the results of this study highlighted a significant change in hand hygiene practices and AHC in the pandemic period. In particular, this study found a consistent and almost three-fold AHC increase at both the hospital and department levels in 2020, followed by a decrease in 2021. Some suppositions can be made on the nature and possible causes of this change. The increase could be related to the collective will to reduce viral transmission. The particular health emergency situation in Italy may have had an impact. In this sense, the pressure on the National Health Service, the hospital and wards conversion for the management of COVID-19 patients, and the expansion and subsequent saturation of intensive care unit beds [19,20,21], could have all contributed to the AHC increase. The AHC decrease in 2021 appears to indicate a progressive readjustment after the greater crisis phase. It is likely that behavioral factors are involved: fear of infection could have led healthcare personnel and patients to initially improve HH practices in the early stages of the pandemic [17,22]; however, with the protracting of the emergency situation, some degree of de-sensitization to the risk posed by infection could have occurred [23,24,25], leading to reduced AHC. AHC values found in 2021 are a source of concern: if this downward trend were to be confirmed in the next years, it could lead to pre-pandemic AHC levels, when various facilities failed or barely succeeded to comply with the minimum requirements determined by the WHO [1,18].

Based on results of this study, it is difficult to estimate the potential impact of the pandemic on AMR rates; however, results appear to show a decreeing MRSA trend. Most importantly, no significant increase in AMR rates was observed, confirming previous predictions [26,27,28,29,30]. Increased adherence to hand hygiene practices during the pandemic may have been a factor in the decreased circulation of resistant microorganisms at the hospital level, in line with previous observations of the protective effect of HH on MRSA transmission [31,32,33,34,35].

Concerning the correlation between AMR rates and AHC, in 2020 and 2021, an inverse proportion between AHC and infection rates was found. However, the only significant result was found between CRE rates and AHC in 2021. It is possible that the introduction of the new mandatory surveillance CSIA (“Consumo di soluzione idroalcolica”) on AHC has resulted in more efficient monitoring and data transmission, and possibly to a positive “surveillance effect” [36]. The significant increase in consumption during the pandemic period could have had a protective effect on HAI transmission; however, other study designs are necessary to investigate this hypothesis.

This study had several limitations. First, there were limitations due to study design: potential confounding factors may have led to the more favorable outcomes in terms of AMR rates seen in pandemic years, such as other preventive measures. Another limitation is the relatively limited number of observations: even though we included data from all public acute-care hospitals in our region, analyses were performed at the health-system level. Furthermore, due the lack of data of MRSA/CRE rates, the correlation with AHC was not performed for all the facilities. It must also be noted that correlation analysis tells us nothing about potential cause and effect relationships between two variables, only that there is a statistical association. Regarding the laboratories analysis, no fecal swab or other samples were studied regarding AMR bacteria. Finally, the presence of very heterogeneous data in a limited time frame certainly affected the cross-correlation results.

## 4. Materials and Methods

### 4.1. Study Design and Data Collection

This was a retrospective study conducted in 27 public hospitals and trusts of Piedmont, Northern Italy. The study period was 5 years, from January 1, 2017 to December 31, 2021.

Each facility’s hospital management provided AHC data from 2017 to 2019 through the regional quality indicator surveillance program. For the years 2020–2021, data were collected through the national surveillance system (CSIA, Consumo di soluzione idroalcolica); however, data sources and methodology for data collection remained the same [18]. AHC data were collected at hospital and department levels, namely general surgery, general medicine, and intensive care units (ICUs). Hospital-level AHC refers exclusively to inpatient consumption for all departments (i.e., not limited to the three abovementioned main department). AHC was expressed as liters per 1000 patient-days (l/1000 pd) and annual medians were considered.

Data on MRSA and CRE were extracted from the AMR regional surveillance system. The regional surveillance system collects bloodstream and cerebrospinal fluid infection data, along with related susceptibility test results, from participating laboratories. The regional AMR surveillance adheres to the protocol and definitions of the European Antimicrobial Resistance Surveillance Network (EARS-net) established by the European Centre for Disease Prevention and Control (ECDC). For the current study, data on the annual median percentage of MRSA and CRE over invasive isolates was considered, which was defined as, respectively, the proportion of oxacillin and cefoxitin-resistant *S. aureus* isolates over all *S. aureus* invasive isolates and of carbapenem-resistant *Acinetobacter* spp., *Escherichia coli*, *Pseudomonas aeruginosa* and *Klebsiella pneumoniae* isolates over respective invasive isolates. MRSA and CRE infections rates were expressed as a percentage (number of positive isolates over total number of isolates).

### 4.2. Statistical Analysis

Median AHC and AMR trends over the considered period were evaluated using descriptive statistics. Data for the years 2017–2019 were compared with 2020 and 2021 by performing Mann–Whitney U tests, due to non-normal distribution using the Shapiro–Wilk test.

Time-repeated Spearman’s correlation was performed to assess the degree of statistical association over time between AHC and MRSA/CRE rates. Two analyses were performed for each year, correlating AHC at the hospital level with overall MRSA and CRE positive rates. For each, a scatter plot was used to identify outlier values that were not considered in correlation analyses. The strength of the association was estimated with the coefficient of determination R² as follows: 0 to 0.19 (very weak correlation); 0.2 to 0.39 (weak correlation); 0.4 to 0.69 (moderate correlation); 0.7 to 0.89 (strong correlation); 0.9 to 1 (very strong correlation).

We performed a pre-whitening of the data, removing the first order autocorrelation from the time series data. This was done by creating a lagged variable and running a linear regression of the original variable (dependent variable) onto the lagged version (independent variable), saving the unstandardized residuals. A cross-correlation between the pre-whitened variables of AHC and MRSA/CRE rates was performed.

Statistical analyses were performed using IBM SPSS Statistics (version 28.0.1.0). The significance level was set at two-tailed *p* < 0.05.

## 5. Conclusions

In conclusion, this study supports the importance of AHC monitoring, which occurred through the establishment of the new CSIA surveillance system. Results of this study suggest that improving AHC is an attainable goal and indicate the magnitude of achievable improvement in our region. Furthermore, our results support the inverse relation between AHC and infection rates with antimicrobial resistant bacteria.

However, other strategies are needed to maintain the high levels of AHC attained during the pandemic, in order to avoid a progressive drop that has already begun in 2021. In this sense, new studies will be needed to better understand the association between the two variables and monitor how the situation will evolve in a post pandemic context.

## Figures and Tables

**Table 1:** Alcohol-based handrub consumption (AHC), and methicillin-resistant Staphylococcus aureus (MRSA) and carbapenem-resistant Enterobacteria (CRE) rates among 27 hospitals in Piedmont, 2017–2021.

	2017–2019	2020	*p*-Value (17–19 vs. 2020) *	2021	*p*-Value (17–19 vs. 2021) *
**AHC (liters/1000 pd), median (IQR)**					
Medical department	11.9 (8.3–14.3)	32.7 (27.3–35.2)	**<0.001**	19.1 (13.6–29.4)	**<0.001**
Surgical Department	10.6 (9.1–15)	21.4 (16.9–23.4)	**0.014**	19.8 (13.8–26)	**<0.001**
Intensive care units	41.1 (33.5–52.2)	90.7 (71.5–130.9)	**0.003**	62.6 (45.3–79)	**0.001**
Overall (hospital level)	12.9 (9–19.3)	29.2 (25.7–36.5)	**<0.001**	24.4 (16.4–34)	**<0.001**
**Antimicrobial resistance rates, median (IQR)**					
MRSA (overall)	38 (32.2–48.3)	38.6 (28.4–50)	0.973	30.4 (21.8–34.6)	**0.013**
CRE (overall)	14 (6.5–29.2)	19.8 (13.8–27.7)	0.430	11.1 (9.4–27.2)	0.974

* Mann-Whitney U tests were used to compare the distribution of the pre-pandemic three-year period (2017–2019) with the following two years, 2020 and 2021, respectively. Significant values are expressed in bold. IQR: interquartile range.

**Table 2:** Correlation between alcohol-based handrub consumption (AHC), and methicillin-resistant Staphylococcus aureus (MRSA) and carbapenem-resistant Enterobacteria (CRE) rates at the hospital level.

	Hospital Level AHC vs. MRSA Rate	Hospital Level AHC vs. CRE Rate
2017 Spearman’s ρ, (N)	−0.10 * (N = 22)	−0.067 (N = 24)
*p*-value	0.964	0.756
R²-value	0.037	0.025
2018 Spearman’s ρ, (N)	−0.193 * (N = 22)	0.094 * (N = 23)
*p*-value	0.389	0.671
R²-value	0.094	0.003
2019 Spearman’s ρ, (N)	0.089 ** (N = 20)	0.177 (N = 23)
*p*-value	0.709	0.432
R²-value	0.008	0.031
2020 Spearman’s ρ, (N)	−0.340 * (N = 22)	−0.215 (N = 20)
*p*-value	0.121	0.362
R²-value	0.116	0.046
2021 Spearman’s ρ, (N)	−0.287 (N = 11)	**−0.646 (N = 11)**
*p*-value	0.392	**0.032**
R²-value	0.083	**0.418**

Significant values are expressed in bold. * Not considering one outlier value, ** not considering two outlier values.

## Data Availability

Data will be made available upon reasonable request.
